# Identifying miRNA-mRNA Integration Set Associated With Survival Time

**DOI:** 10.3389/fgene.2021.634922

**Published:** 2021-06-29

**Authors:** Yongkang Kim, Sungyoung Lee, Jin-Young Jang, Seungyeoun Lee, Taesung Park

**Affiliations:** ^1^Department of Statistics, Seoul National University, Seoul, South Korea; ^2^Center for Precision Medicine, Seoul National University Hospital, Seoul, South Korea; ^3^Department of Genomic Medicine, Seoul National University Hospital, Seoul, South Korea; ^4^Department of Surgery and Cancer Research Institute, Seoul National University College of Medicine, Seoul, South Korea; ^5^Department of Mathematics and Statistics, Sejong University, Seoul, South Korea; ^6^Interdisciplinary Program in Bioinformatics, Seoul National University, Seoul, South Korea

**Keywords:** statistical method, miRNA-mRNA integration, personalized medicine, pancreatic ductal adenocarcinoma, The Cancer Genome Atlas

## Abstract

In the “personalized medicine” era, one of the most difficult problems is identification of combined markers from different omics platforms. Many methods have been developed to identify candidate markers for each type of omics data, but few methods facilitate the identification of multiple markers on multi-omics platforms. microRNAs (miRNAs) is well known to affect only indirectly phenotypes by regulating mRNA expression and/or protein translation. To take into account this knowledge into practice, we suggest a miRNA-mRNA integration model for survival time analysis, called *mimi-surv*, which accounts for the biological relationship, to identify such integrated markers more efficiently. Through simulation studies, we found that the statistical power of *mimi-surv* be better than other models. Application to real datasets from Seoul National University Hospital and The Cancer Genome Atlas demonstrated that *mimi-surv* successfully identified miRNA-mRNA integrations sets associated with progression-free survival of pancreatic ductal adenocarcinoma (PDAC) patients. Only *mimi-surv* found miR-96, a previously unidentified PDAC-related miRNA in these two real datasets. Furthermore, *mimi-surv* was shown to identify more PDAC related miRNAs than other methods because it used the known structure for miRNA-mRNA regularization. An implementation of *mimi-surv* is available at http://statgen.snu.ac.kr/software/mimi-surv.

## Introduction

MicroRNAs (miRNAs) are small, non-coding RNAs that function to regulate target messenger RNAs (mRNAs), based on sequence complementarity. It is well known that miRNAs affect nearly all developmental and pathological processes in animals, particularly in cell development, and many cancer types are affected by miRNA regulation by downregulating their target mRNAs (Ha and Kim, 2014).

Using a well-known regulation mechanism, many studies have focused on finding the target mRNAs. The biological context of regulation mechanism between miRNA and target mRNA can be easily explained by showing significant negative correlation between them and investigating their relationship with the phenotypes (Enerly et al., 2011; Xu et al., 2019). For instance, hierarchical clustering on miRNA expression profiles found that the expression levels of the tumor suppressor gene, *TP53* are associated with specific clusters (Enerly et al., 2011). When the number of target genes is small, this approach is effective. However, it is more difficult to identify novel combinations of miRNA and its target mRNAs that are concurrently associated to the phenotype.

To perform an integrated analysis of miRNA and its target mRNAs, two-step analysis has been commonly used in many studies. The first step chooses miRNAs associated with specific phenotypes. The second step further investigates expression levels of known target mRNAs that are negatively correlated with each miRNA (Enerly et al., 2011; Yonemori et al., 2017). However, this approach only focuses on the relationship between phenotypes and miRNAs without providing information about how miRNAs and their inhibited mRNAs affect observed phenotype together.

On the other hand, a hierarchical structured component analysis of miRNA-mRNA integration (*HisCoM-mimi*) has been recently proposed to investigate how miRNAs indirectly affect the phenotype with biological relationships between the miRNAs and their target mRNAs [5; 6]. *HisCoM-mimi* is a component-based method that models biological relationships as hierarchically structured “components,” to efficiently identify miRNA-mRNA integration sets. *HisCoM-mimi* has an advantage of handling many types of phenotypes from an exponential family distribution under the framework of a generalized linear model. While its application to cancerous vs. normal tissues successfully identified more biologically plausible and intuitive interpretations than other methods (Kim et al., 2018), it cannot be applicable to the survival analysis which is one of prominent interest among the cancer studies.

In this study, we propose a hierarchical structured component analysis of miRNA-mRNA integration to survival phenotype, called *mimi-surv* using a Cox Proportional Hazard (Cox-PH) model (Cox, 1972; Kim, 2018; Kim et al., 2018). Like *HisCoM-mimi*, *mimi-surv* is also a component-based analysis, such as pathway models we developed for rare variant pathway analysis (Lee et al., 2016, 2019). In this respect, the proposed model introduces a latent variable for each miRNA and its target mRNAs as a component and fits one augmented model including all latent variables to determine the associations with the survival phenotype.

We applied the proposed approach, *mimi-surv*, to two real datasets from pancreatic ductal adenocarcinoma (PDAC) patients. It is noted that PDAC is one of the most lethal gastrointestinal malignancies. Despite improvements in perioperative outcomes, PDAC has a poor prognosis, with a 5-year survival rate of only 6%, worldwide (Greither et al., 2010). Because most patients are diagnosed in the advanced stages, and effective systemic therapies are lacking. Consequently, many researchers have focused on developing novel prognostic markers of PDAC. For example, several studies have identified cell-free miRNAs as prognostic markers of PDAC among which high expression of *miR-21* was shown to have a significant effect on overall survival time (Frampton et al., 2015). We considered two real PDAC datasets; one is a microarray-based dataset from PDAC patients from Seoul National University Hospital (SNUH), and the other is high-throughput sequencing data, obtained from The Cancer Genome Atlas (TCGA). From those datasets, we tried to find prognostic factors for survival after surgery of PDAC by survival analysis on integrated miRNA-mRNA sets, using *mimi-surv*.

In spite of that some prognostic miRNAs have been identified, their precise roles in the progression of PDAC have not been easy to interpret due to absence of overall grasp of vast network of miRNA-mRNA interaction. In this article, we demonstrated how well our hierarchical component-based approach can embrace such a biological concept. Moreover, the proposed *mimi-surv* was compared with many other survival analysis methods throughout the simulation studies.

## Materials and Methods

### The Mimi-Surv Model

**FIGURE 1:**
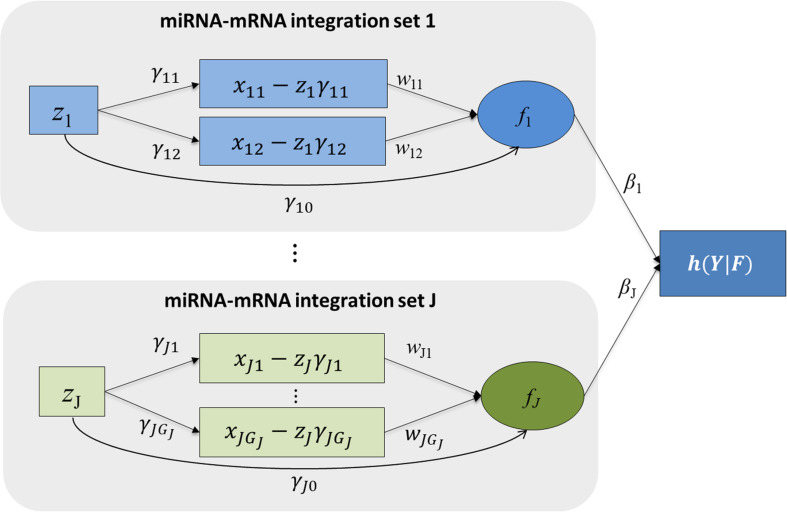
Schematic diagram of *mimi-surv* model. Rectangles and circles indicate observed and latent variables, respectively. Arrows indicate conceptualized directions of effects between the variables. Each miRNA-mRNA integration set consists of one miRNA and its target mRNAs. Each miRNA-mRNA integration set *j* is summarized by the latent variable *f*_*j*_ which is linear combination of *z*_*j*_ and its adjusted mRNA expressions.

Figure 1 shows the schematic plot for *mimi-surv* model. For survival data analysis, the Cox-PH model is used (Cox, 1972). miRNA-mRNA integration set contains the miRNA, mRNA affected by the miRNA, and miRNA integration latent variable. The miRNA-mRNA integration set shows that the miRNA’s direct and indirect effects on the phenotype are coming from target mRNAs. Each miRNA-mRNA integration set consists of one miRNA (*z*_*ij*_), and mRNAs (*x*_*ij*__1_, *x*_*ij*__2_, …, *x*_*ijGj*_) which were regulated by the miRNA. miRNA-mRNA integration set *j* is summarized by the latent variable *f_*ij*_* which is a linear combination of *z*_*ij*_ and *x*_*ij*__1_, *x*_*ij*__2_, _…_, *x*_*ijGj*_. Thus, the effect of miRNA-mRNA integration set *j* on the hazard rate is computed by *β_*j*_*. Detailed fitting approaches for *mimi-surv* are described as follows.

### Adjusting mRNA Expression by miRNA Regulation Information

The *mimi-surv* model consists of three parts. First, the miRNA-mRNA part estimates effect of miRNA on target mRNAs. Second, the miRNA integration latent part models overall effect of each miRNA. Finally, the phenotype-latent part associates all latent variables with the target phenotype. In the miRNA-mRNA part, a simple linear combination relationship is constructed between miRNA and target mRNAs, as shown in the following Equation 1:

(1) $${\hat{X}}_{i\,j\,k}=x_{i\,j\,k}-\gamma_{j\,k}\,z_{i\,j},i=1,\cdots ,N,j=1,\cdots ,J,k=1,\cdots ,G_{j},$$

where *x*_*ijk*_ is the *i*^*th*^ individual’s mRNA expression of the *k*^*th*^ gene, which is inhibited by *j*^*th*^ miRNA, *z_*i*__*j*_* is the *i*^*th*^ individual’s *j*^*th*^ miRNA expression, *γ_*jk*_* is the inhibition coefficient for the *j*^*th*^ miRNA for the *k*^*th*^ gene, and *G*_*j*_ is the number of inhibited mRNAs by the *j*^*th*^ miRNA. By estimating the miRNA inhibition coefficients *γ_*jk*_*, the *k*^*th*^ gene’s mRNA expression after adjusting the inhibition effect of the *j*^*th*^ miRNA can be obtained.

### Latent Structures

The proposed *mimi-surv* models an aggregated effect of both miRNA and mRNA as a latent variable *f*_*ij*_. As defined in Equation 2, the latent variable *f*_*ij*_ represents the global effect of the miRNA’s activity, as measured by a linear combination of both the inhibition effects (*w*_*jk*_) of its target mRNA(s) expression and the direct effect (*γ_*j*_*_0_) of the miRNA expression value.

(2) $$f_{i\,j}=\gamma_{j\,0}\,z_{i\,j}+\sum_{k=1}^{G_{j}}{\hat{X}}_{i\,j\,k}\,w_{j\,k}$$

The latent variables are finally associated to the target phenotype using a Cox-PH model (Cox, 1972) as shown in Equation 3, under the assumption that the hazard rate is proportional to the risk factors over time.

(3) $$h\,\left(y_{i}|F_{i}\right)=h_{0}\,\left(y_{i}\right)\,\exp\left(\sum_{j=1}^{J}\left[\gamma_{j\,0}\,z_{j}+\sum_{k=1}^{G_{j}}{\hat{X}}_{i\,j\,k}\,w_{j\,k}\right]\,\beta_{j}\right)=h_{0}\,\left(Y\right)\,\exp\left(\sum_{j=1}^{J}f_{i\,j}\,\beta_{j}\right),$$

where *y*_*i*_ denotes the survival time, *Y* denotes the vector of *y*_*i*_, and *h*(*y*_*i*_|*F*) denotes the hazard function of the *i*^*th*^ sample. In addition, *h*_0_(*Y*) is a baseline hazard function, and *β_*j*_* represents the effect of *f*_*ij*_ on the hazard rate, as a risk factor. Then, the partial likelihood function, *L*_*p*_, is defined as follows:

$$L_{p}=\frac{\prod_{i:C_{i}=1}\exp\left(\sum_{j=1}^{J}f_{i\,j}\,\beta_{j}\right)}{\sum_{q:y_{q}=y_{i}}\exp\left(\sum_{j=1}^{J}f_{q\,j}\,\beta_{j}\right)},$$

(4) $$C_{i}=\left\{ \begin{array}{c} 0\,\left(i^{\text{th}}\,\mathrm{individual}\,\mathrm{is}\,\mathrm{censored}\right)\\ 1\,\left(i^{\text{th}}\,\mathrm{individual}\,\mathrm{is}\,\mathrm{deceased}\right) \end{array}\right.$$

### Model Fitting

In model fitting, we estimate the parameters of *mimi-surv* by adopting the algorithm of *HisCoM-mimi* which is based on the alternating least squares (ALS) algorithm for the penalized log-likelihood function, with penalty parameters (Kim et al., 2018). In the *mimi-surv* model, the objective function to be maximized is expressed as follows:

(5) $$\phi =\sum_{i:c_{i}=1}\left(\sum_{j=1}^{J}f_{i\,j}\,\beta_{j}-\log\,\sum_{q:y_{q}=y_{i}}\exp\left(\sum_{j=1}^{J}f_{q\,j}\,\beta_{j}\right)\right)-\frac{1}{2}\,\lambda_{m}\,\sum_{j=1}^{J}\sum_{k=1}^{G_{j}}P_{\lambda_{m\,m}}\,\left(w_{j\,k}\right)-\frac{1}{2}\,\lambda_{m\,m}\,\sum_{j=0}^{J}P_{\lambda_{m}}\,\left(\beta_{j}\right).$$

Here, the first sum consists of the partial likelihood from a Cox-PH model and the remaining term consists of two penalization parts with tuning parameters of *λ_*m*_* and *λ_*mm*_*. These two *λ*s are so-called the tuning parameters of both the miRNA-mRNA pairs and the integrated latent components to adjust the strength of the penalty function (Cox, 1972). *P*_*λ*_*mm*__ and *P*_*λ*_*m*__ denote penalty functions for *w* and β, respectively. Any regularization function can be used. For example, for β it can be defined as $\sum_{j=1}^{J}\beta_{j}^{2}$ for ridge,$\sum_{j=1}^{J}\left|\beta_{j}\right|$ for lasso, and $\left(\frac{1}{2}\,\sum_{j=1}^{J}\beta_{j}^{2}+\sum_{j=1}^{J}\left|\beta_{j}\right|\right)$ for Elastic-Net.

We used the ALS algorithm to maximize the objective function by the two-step algorithm. The first part of the ALS algorithm is maximizing the objective function, *ϕ*, with the conditioning set of *f*_*qj*_, and finding solutions for a set of *β_*j*_*. The second part of algorithm is, maximizing the objective function, with a conditioning set of *β_*j*_*, as calculated in the previous step, and updating the set of *f* values. Then these two steps are iterated until the solution is converged.

In the *mimi-surv* model, *β_*j*_* indicates the effect size of *j*^*th*^ miRNA-mRNA integration set and *w*_*jk*_ indicates the effect size of *k*^*th*^ mRNA inhibited by *j*^*th*^ miRNA. In this study, we find the significant integrated effects of miRNA and its inhibited mRNAs, and we used *mimi-surv* to test *β_*j*_*, which summarized mRNA-miRNA integration set.

We performed a simple permutation scheme to test the statistical significance of *β_*j*_* and computed *p*-values and their *q*-values for the multiple testing adjustment (Ma et al., 2014). The number of permutations was set to 1,000. However, it can be increased easily to improve the accuracy of *p*-values. If one of the penalty functions is pre-specified, *mimi-surv* provides the corresponding *p*-values. However, if the choice of a penalty function is not given, *mimi-surv* can use a simple approach that picks the maximum estimate from multiple penalties, namely *maxT*. Through permutations, the null distribution of *maxT* is generated from which the *p*-value can estimated.

### Comparative Models

We compared the performance of *mimi-surv* with various types of Cox-PH models, including a single miRNA Cox-PH model (single) and multiple penalized Cox-PH regression models with different penalties such as ridge, lasso, Elastic-Net (*EN*), and group lasso (*grplasso*) (Lee and Silvapulle, 1988; Tibshirani, 1996; Zou and Hastie, 2005; Meier et al., 2008)]. The objective function for multiple penalized Cox-PH model is given as follows:

(6) $$\phi_{1}=\sum_{i:c_{i}=1}\left(\sum_{j=1}^{J}\delta_{j}\,z_{i\,j}-\log\,\sum_{q:y_{q}\leq y_{i}}\exp\left(\sum_{j=1}^{J}\delta_{j}\,z_{q\,j}\right)\right)-P_{\theta }\,\left(\delta_{j}\right),$$

where *P*_θ_(δ_*j*_) denotes regularization function, which can be defined as $\theta \,\sum_{j=1}^{J}\delta_{j}^{2}$ for ridge,$\theta \,\sum_{j=1}^{J}\left|\delta_{j}\right|$ for lasso, and $\theta \,\left(\frac{1}{2}\,\sum_{j=1}^{J}\delta_{j}^{2}\,\sum_{j=1}^{J}\left|\delta_{j}\right|\right)$ for *EN*. Here θ is the tuning parameter to adjust the strength of the penalty function.

For a *grplasso* Cox-PH model (Meier et al., 2008), using the group information from the miRNAs and mRNAs, the following regression model is given:

$$h\,\left(Y\right)=h_{0}\,\left(Y\right)\,\exp\left(\sum_{j=1}^{J}\delta_{j}\,z_{j}+\sum_{j=1}^{J}\sum_{k=1}^{G_{j}}\lambda_{j\,k}\,{\hat{x}}_{j\,k}\right),$$

(7) $$\mathrm{subject}\,\mathrm{to}\,\left(\left|\delta_{j}\right|+\sum_{k=1}^{G_{j}}\left|\lambda_{\mathrm{jk}}\right|\right)\geq t.$$

To find the optimal tuning parameter *θ*, we performed 10-fold cross-validation and then determined the value of *θ*, which minimizes the value of the objected function for the validation set.

### SNUH and TCGA Datasets

**FIGURE 2:**
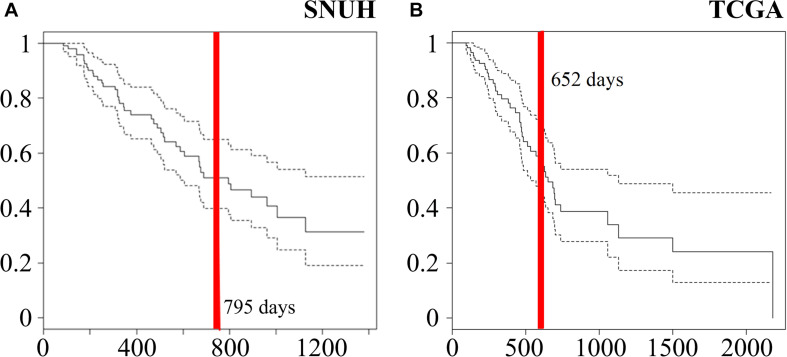
Kaplan-Meier curves of **(A)** 95 samples of SNUH dataset and **(B)** 112 samples of TCGA dataset. Red vertical lines indicate median survival times (795 and 652 days SNUH and TCGA, respectively).

The SNUH dataset consists of 95 PDAC patients in which the average of age was 65.2 years with a standard deviation 9.4 years. There were 46 male and 49 female patients. The median survival time after surgery was 795 days, which is indicated by a red vertical line in a Kaplan-Meier plot as shown in Figure 2A.

mRNA expression data was produced by the Human Gene 1.0 ST array (Affymetrix, Santa Clara, CA, United States). For background correction, the expression values were processed by Robust Multi-array Averaging (RMA), using the Affymetrix console, followed by quantile normalization. For the same patient, miRNA expression was obtained from the GeneChip miRNA 3.0 array (Affymetrix, Santa Clara, CA, United States). miRNA expression values were normalized by RMA, and only the human-derived miRNA targets were selected. The normalization of the background correction of the *j*^*th*^ human probe of the *i*^*th*^ sample (*x*_*ij*_) was done using the other species’ probes as background intensities as shown in Equation 8.

(8) $$x_{i\,j\,\left(\text{norm}\right)}=x_{i\,j}-\text{median}\left(x_{i\,j},j\in \mathrm{non}-\mathrm{human}\mathrm{miRNA}\right)$$

On the other hand, TCGA PDAC dataset were downloaded from the Genomic Data Commons (GDC) data portal of the U.S. National Cancer Institute^1^ (Cancer Genome Atlas Research Network, Weinstein et al., 2013). To normalize mRNA-seq and miRNA-seq datasets, Fragments Per Kilobase Million (FPKM) was measured for each read count. For miRNA expression profiling, Illumina HiSeq (Illumina Inc., San Diego, CA, United States) was used. We collected 185 TCGA PDAC data sample for analysis. The read counts were log-transformed after adding a pseudo count of 0.5. In survival analysis, we excluded 25 non-PDAC samples and 47 PDAC samples whose follow-up time was less than 3 months because the cause of their deaths is not clear. After excluding these cases, we have 112 samples that consist of 48 males and 64 females. The mean age was 63.9 years with a standard deviation 11.1 years. Furthermore, the median survival time was 585 days as indicated by a red vertical line in a Kaplan-Meier plot in Figure 2B.

### Identification of miRNA-mRNA Integration Set

For miRNA-mRNA integration analysis, we generated miRNA-mRNA integration sets which collected miRNAs and their target mRNAs satisfying two conditions as follows: (i) Reported target mRNAs by sequence-based target prediction results from TargetScan 7.1 (Agarwal et al., 2015) and (ii) significant negative correlation coefficients between miRNAs and mRNAs from SNUH dataset.

From the miRNA-mRNA pairs from TargetScan using SNUH dataset, we calculated Pearson’s correlation and performed one-sided *t*-test to select the pairs with significant (*p*< 0.05) negative correlation. For those using TCGA dataset that contains many zero read counts, we first filtered out spurious pairs of miRNA-mRNA by performing one-sided *t*-test to test whether the average mRNA expression of the samples with zero miRNA read count was larger than that of the samples with non-zero miRNA read counts (*p* < 0.05). For those significant pairs, we then tested whether a correlation between target mRNAs and miRNAs was less than 0, using the samples with nonzero miRNA read counts.

### Simulation Study and Real Data Analysis

To compare which method had a better power to discover the true signal miRNA-mRNA pair, we performed simulation studies to compute type I errors and power of *mimi-surv* and the compared methods, using the miRNA expression values of the SNUH PDAC dataset that consists of 64 miRNAs and 6,226 significant miRNA-mRNA pairs. Among those miRNA-mRNA pairs, we selected two, five and ten causal miRNAs to simulate phenotypes. Table 1 lists those miRNAs and their regulated mRNAs. To generate a simulation dataset, we used the same simulation settings as we did for our previous *HisCoM-mimi* analysis (Kim et al., 2018).

**TABLE 1:** List of causal miRNAs and the numbers of target mRNAs used in simulation.

miRNA	# target mRNAs	Regulated mRNAs in SNUH data
*miR-212*^1,2,3^	425	PAX5, SHISA9
*miR-219*^1,2,3^	445	HMGA2, EGR3
*miR-200b*^2,3^	9	SLIT2, BNC2, CDH11
*miR-32*^2,3^	172	PRKAB2, SNX2
*miR-362*^2,3^	125	PLAT, SMAD2, CHRDL1
*miR-204*^3^	56	GRIN2B, HMGA2, ARNTL2, ACADL, TDRD6
*miR-217*^3^	449	LHX1, NR4A2, PKP1, SHOX, TRIM71, CAMK2A
*miR-1297*^3^	285	MCL1, RLF, RAB5IF, EDEM3
*miR-496*^3^	149	FLRT2, PAX6, SDHC, SERAC1, SYT5, UBXN2A
*miR-670*^3^	550	FRAS1, ANKRD50, LIN28B, PDE7A, SLC4A4, TP53INP1, TRIB2, CD248

*^1^miRNAs used in the simulation with two causal miRNAs. ^2^miRNAs used in the simulation with five causal miRNAs. ^3^miRNAs used in the simulation with ten causal miRNAs.*

We assumed a true model for generating simulated phenotype, as given in Equation 9. We considered that all causal miRNA-mRNA sets, having an effect size of *β*. Also, we considered regulated target mRNAs of the miRNA-mRNA sets, having the common effect size, *w*_11_ = *w*_1_*_*p*_*, and their regulating miRNA having the effect size *γ*_10_. We then considered three scenarios with different number of causal miRNAs (2, 5, and 10). For the scenario with two causal miRNAs, *miR-212* and *miR-219* were used to generate phenotypes. In the scenario with five causal miRNAs, *miR-200*, *miR-32*, *miR-362* were considered, in addition to the aforementioned two miRNAs. Lastly, five miRNAs (*miR-204*, *miR-217*, *miR-1297*, *miR-496*, *miR-670*) were additionally used in the scenario with ten causal miRNAs (see Table 1 and section “Results”). The statistical powers were computed as the proportion of replicates whose empirical *p*-values of causal miRNAs are nonzero and significant.

(9) $$h\,\left(Y|X,Z\right)=h_{0}\,\left(Y\right)\,e\,x\,p\,\left(\beta \,\left(\gamma_{10}\,z_{1}+\sum_{k=1}^{K}w_{1\,k}\,{\hat{x}}_{k}\right)\right)$$

In the real data analysis, to deal with the multiple testing problem, we used Benjamini-Hochberg procedure to calculate False Discovery Rate (FDR) and calculated the *q-*value. The threshold of *q*-value was set to 0.1.

## Results

### miRNA-mRNA Pairs Extraction

We first extracted miRNA-mRNA pairs using the SNUH and TCGA datasets. For the SNUH dataset, TargetScan provided 370,075 pairs of miRNA-mRNA for 503 unique miRNAs. Our filtering strategy (see Methods) narrowed down the initial 370,075 set of pairs to 6,226 pairs that resulted in 54 unique miRNAs. For the TCGA dataset, TargetScan provided 51,014 pairs of miRNA-mRNA for 69 unique miRNAs. Unlike SNUH microarray dataset, we found that only 133 pairs of miRNA-mRNA from nine unique miRNAs were left when Pearson correlation tests were used. As noted in the Methods, the two-side filtering step resulted in 1,456 pairs with 23 unique miRNAs having at least one significant mRNA.

While two datasets showed generally concordant patterns of miRNA-mRNA selection as shown in Table 2, the number of mRNAs in each integration set has dataset-specific patterns. While *miR-211* integration set has the greatest number of overlapped mRNAs when combining those of SNUH and TCGA, the greatest number from each of SNUH and TCGA was *miR-141* and *miR-133b*, respectively.

**TABLE 2:** The number of mRNAs included in the miRNA-mRNA integration set.

miRNA	# overlapped	# mRNAs (SNUH)	# mRNAs (TCGA)
*miR-105*	41	331	51
*miR-133b*	3	10	281
*miR-141*	28	469	37
*miR-192*	1	47	1
*miR-200b*	2	4	9
*miR-200c*	10	336	15
*miR-206*	8	50	114
*miR-211*	60	461	119
*miR-372*	7	24	207
*miR-429*	3	32	14
*miR-488*	13	43	62
*miR-524*	4	50	17
*miR-670*	2	8	131
*miR-96*	3	36	43

### Simulation Results

**FIGURE 3:**
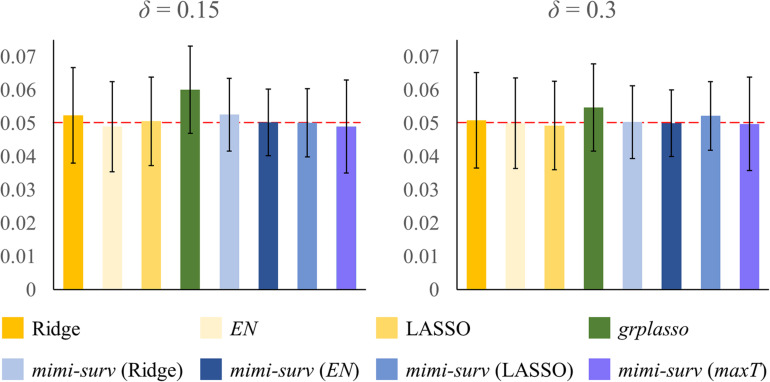
Result of type I error evaluation. Bars indicate estimated type I error rate with given parameters (censoring fraction *δ*). Note that the type I errors were evaluated by fixing all parameters to 0.

The simulation was conducted using the SNUH dataset with 54 miRNAs and their 6,226 miRNA-mRNA pairs, with the following parameters: two censoring fractions (*δ* = 0.15 and 0.3), three miRNA effect sizes (*γ* = 0.2, 0.3, and 0.4), three mRNA effect sizes (*w* = 0.5, 0.6, and 0.7). Effect of miRNA-mRNA integration set *β* was fixed to 1 for simplicity. The significance level *α* was set to 0.05. First, we estimated the type I error of each method by setting all parameters to 0 with the censoring fraction as *δ*. As shown in Figure 3, type I errors were controlled at *α* = 0.05 in all models, except *grplasso* (Meier et al., 2008) model which showed slightly inflated type I errors. In addition, *mimi-surv* models generally showed slightly smaller standard deviations of type I errors than the compared methods (±0.009∼0.01 for *mimi-surv*,±0.013∼0.014 for the other models). Note that the type I errors of both *mimi-surv* and the compared methods were not affected by the zero proportion of miRNA expression (zero proportion 10, 30, and 50%). In addition, we also checked an effect of penalty selection in the simulation. Since the selection of optimal penalty is challenging in Cox-PH regression (Benner et al., 2010; Ojeda et al., 2016), we applied a simple strategy that combines the three penalties by selecting the maximum of the estimates from three different penalties (lasso, ridge, and *EN*), namely *maxT*. Simulation results showed that *mimi-surv* with the proposed *maxT* approach successfully controlled type I errors with significance level of 0.05 (0.049 ± 0.014 for *mimi-surv*), as shown in Figure 3.

**FIGURE 4:**
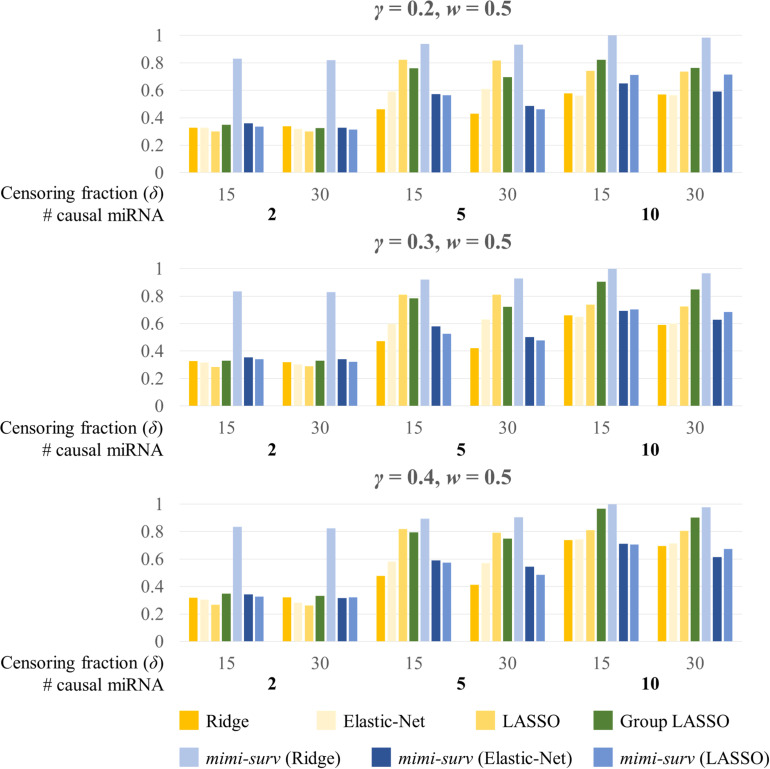
Statistical powers of *mimi-surv* and the compared methods with different miRNA effect sizes (*γ* = 0.2, 0.3, and 0.4). The phenotypes were generated from two, five and ten causal miRNA-mRNA integration set and censoring fraction of 0.15 and 0.3.

Second, we assessed the statistical powers of seven methods (*mimi-surv* with three different penalties, *grplasso*, *lasso*, *ridge*, and *EN*). Here, we generated 200 replicates of simulated phenotypes to assess the power. When variable selection methods (lasso, *EN*, *grplasso*, *mimi-surv* with lasso, and *EN* penalties) produced zero coefficients, their effects were regarded as non-significant. Figure 4 depicts statistical powers of the compared methods with different miRNA effect sizes (0.2, 0.3, and 0.4) and two censoring fractions (0.15 and 0.3). Note that other non-causal miRNAs or mRNAs were also included to the analysis, but they actually did not contribute to the phenotypes at all. In this case, *mimi-surv* with ridge penalty and *grplasso* showed the first and second largest powers, regardless of the miRNA effect sizes. Lasso, *EN*, *mimi-surv* with *EN* and lasso penalties had smaller power than the other methods. While the powers generally increased with the miRNA effect size, their ranks vary widely (Figure 4). Higher censoring rate yielded generally lower power. Note that those tendencies were maintained even if *γ*, *w*, or the number of connected mRNAs were changed.

**FIGURE 5:**
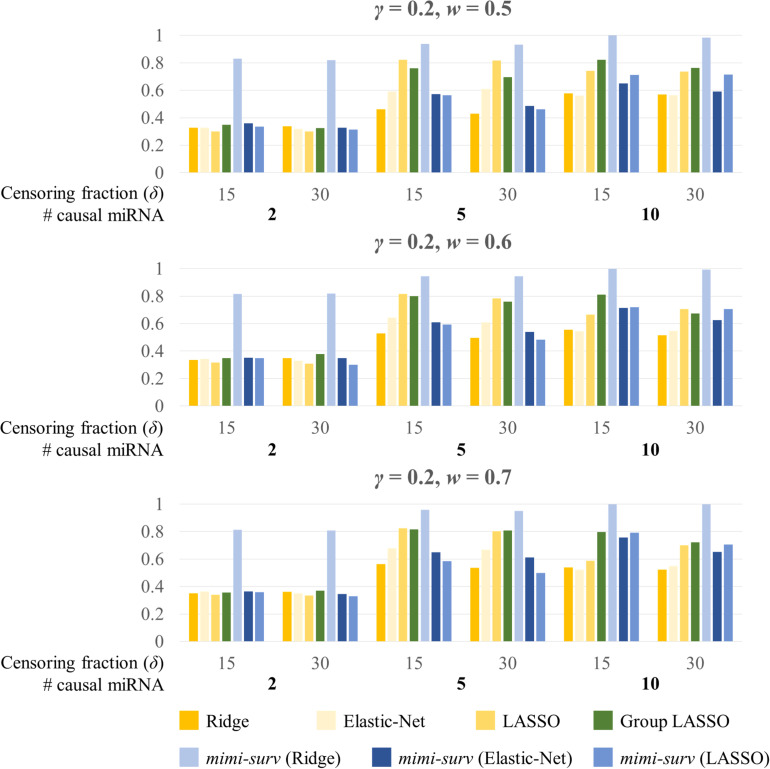
Statistical powers of *mimi-surv* and the compared methods with different mRNA effect sizes (*w* = 0.5, 0.6, and 0.7). The phenotypes were generated from two, five and ten causal miRNA-mRNA integration set and censoring fraction of 0.15 and 0.3.

Figure 5 shows the barplots comparing the power of each method for a fixed miRNA effect size (*γ* = 0.2) and various mRNA effect sizes with censoring fractions of 0.15 and 0.3. Similarly, *mimi-surv* with ridge penalty showed the largest power. Unlike the results from Figure 4, *mimi-surv* with *EN* and lasso showed comparable power to *grplasso* when the number of causal miRNA increases. The same tendency was observed for various values of *γ* and *w*. In addition, the power differences between the results from various values of *γ* and *w* were small.

### SNUH Dataset Analysis Result

In order to identify miRNA-mRNA integration sets, 54 miRNA-mRNA integration sets were selected to which *mimi-surv* along with other methods was applied to identify significant miRNA-mRNA integration sets. In this analysis, we focused on comparing the lists of significant miRNAs obtained from single, ridge, lasso, *EN*, *grplasso*, and *mimi-surv* (Lee and Silvapulle, 1988; Tibshirani, 1996; Zou and Hastie, 2005; Meier et al., 2008).

**FIGURE 6:**
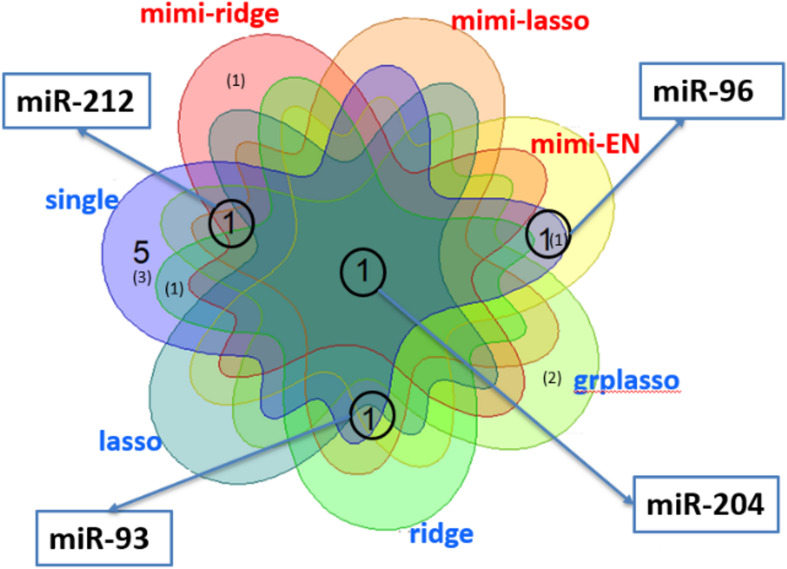
Venn diagram for the number of miRNAs detected by each method in analysis of PDAC data from SNUH. The numbers without brackets show the numbers of miRNAs found in other PDAC analyses, while those within brackets show the number of miRNAs not previously identified.

Figure 6 shows a Venn diagram displaying the number of miRNAs identified by each method, in which the number without brackets shows the number of miRNAs reported in other studies, and those within brackets show the total number of miRNAs found significant by each method. Note that the largest number of miRNAs was detected by single marker analysis. Interestingly, about half (6 out of 14) overlapped with other methods. Of these, *mimi-surv* detected a total of six miRNAs, in which four miRNAs were reported in other PDAC analyses (Ma et al., 2014; Tanaka et al., 2014; Debernardi et al., 2015; Li et al., 2015; Cheng et al., 2017). In general, the penalized Cox-PH methods identified relatively fewer miRNAs than other methods, but ridge penalty had the largest detection rate. Note that all methods commonly detected *miR-204*, which is known for the differential expression relationship between PDAC stage I and stage II-IV samples (Debernardi et al., 2015). In addition, *miR-204* has been used to distinguish solid pseudo-papillary tumors from pancreatic malignancies (Li et al., 2015).

### TCGA Dataset Analysis Result and Comparison

For the analysis of TCGA data, 23 miRNA-mRNA integrations pairs were constructed. Table 2 shows information for the miRNAs detected in the TCGA dataset analysis. For the TCGA data analysis, all the compared methods including single marker analysis and penalized regression methods failed to identify any significant miRNAs. However, *mimi-surv* detected five significant miRNAs with their significant genes, using various types of penalties. Among those results, we successfully replicated one miRNA *miR-96*, which was identified in the analysis of SNUH dataset. *miR-96* is a well-known marker as a suppressor of the KRAS signaling pathway (Tanaka et al., 2014). Among our detected miRNAs, *miR-200c*, *miR-506*, and *miR-96* were previously reported in other PDAC studies (Mees et al., 2010; Bryant et al., 2012; Tanaka et al., 2014; Cheng et al., 2016; Pan et al., 2018; Zhuo et al., 2018).

Table 3 lists the significant miRNAs and their significant target mRNAs detected by *mimi-surv* from both datasets. Interestingly, using the proposed *maxT* approach, *mimi-surv* successfully identified two significant miRNAs (*miR-96* and *miR-133b*) after the multiple testing adjustment (FDR *q*-value < 0.05), and one of those miRNAs (*miR-96*) was the replicated miRNA. In addition, our approach successfully showed the advantage of penalization approach. For instance, *miR-93* has more than 901 target mRNAs, therefore the significance level after multiple testing adjustment can be dramatically small. However, only 7 mRNAs were found significant by *EN*, and only 9 mRNAs were found significant by lasso. As a result, by using *mimi-surv*, we could reduce the number of candidate miRNA-mRNA sets.

**TABLE 3:** Results of statistically significant miRNA and its significant mRNAs from both datasets using *mimi-surv*.

	miRNA	# mRNAs	# significant mRNAs (names)	*β_*mimi*_*	*p*_*mimi*_	*q*_*mimi*_	*Penalty*
S N U H	*miR-204*	5	*N/A*	–0.018	0.015	0.690	Ridge
			1 (GRIN2B)	–0.179	0.004	0.221	Lasso
			1 (GRIN2B)	–0.142	0.031	0.490	*EN*
			*N/A*	–0.179	0.021	0.382	*maxT*
	*miR-93*	901	9	–0.406	0.012	0.319	Lasso
			7	–0.544	0.003	0.178	*EN*
			*N/A*	–0.544	0.005	0.259	*maxT*
	*miR-212*	2	1 (PAX5)	0.015	0.045	0.690	Ridge
			1 (PAX5)	0.008	0.033	0.601	Lasso
	***miR-96***	**34**	**2 (GPM6B, EPHA3)**	**0.209**	**0.017**	**0.462**	***EN***
			***N/A***	**0.209**	**0.020**	**0.382**	***maxT***
	*miR-497*	189	2 (LRRC14, PHF13)	–0.252	0.036	0.490	*EN*
			*N/A*	–0.252	0.046	0.620	*maxT*
	*miR-339*	46	*N/A*	0.024	0.045	0.690	Ridge
T C G A	*miR-133b*	281	2 (ELFN1, KCNJ12)	0.679	0.010	0.218	*EN*
			*N/A*	0.679	0.002	0.044	*maxT*
	*miR-200c*	15	2 (BASP1, LPAR1)	0.131	0.038	0.154	Lasso
			*N/A*	0.131	0.029	0.167	*maxT*
	*miR-506*	109	2 (OXSR1, RAB43)	0.023	0.040	0.249	Ridge
	*miR-206*	115	*N/A*	0.018	0.018	0.142	*maxT*
	***miR-96***	**43**	**2 (FRMD4A, SH3BP5)**	**0.419**	**0.021**	**0.244**	***EN***
			***N/A***	**0.419**	**0.004**	**0.046**	***maxT***

*The replicated miRNA (*miR-96*) has embolden, and the significant mRNAs after the multiple testing adjustment (*miR-96* and *miR-133b*) has underlined.*

## Discussion

In this study, we proposed *mimi-surv* which is a novel approach to identifying significant miRNA-mRNA sets associated with survival time, reflecting the nature of biological process between miRNA and mRNA. The objective of our analysis is to propose an integrative method for using an additional information of mRNA to the analysis of miRNA. Thus, we investigated how much the integrative analysis of miRNAs and mRNAs performs better than the other integrative methods using both miRNAs and mRNAs and the model using only miRNAs.

Through simulation studies, we compared the performance of mimi-surv, with various methods such as a single Cox-PH model, penalized Cox-PH methods with ridge, lasso, *EN* penalties and *grplasso*, including selection of optimal penalties. From the simulation results, it was shown that *mimi-surv* with ridge penalty outperformed other methods, in terms of the statistical power. The analysis of two real datasets of PDAC patients from SNUH and TCGA on which *mimi-surv* showed superior performance in identifying miRNA-mRNA integration sets for survival time. Moreover, *mimi-surv* successfully replicated one miRNA (*miR-96*) from TCGA dataset with statistical significance (*q*-value < 0.01), despite difference of the generation platform (Affymetrix chip vs. Illumina sequencing).

Our study remains with some limitations. First, although our simulation study based on the real SNUH dataset and simulated phenotypes showed that performance of *mimi-surv* with ridge penalty had better power than other penalties, *mimi-surv* with *maxT* approach or *EN* penalty detected more miRNAs in real PDAC data analysis. It is well known that selection of optimal penalty is challenging for Cox-PH model (Benner et al., 2010; Ojeda et al., 2016). For real data application, we recommend trying all applicable penalties to the dataset and select the penalty with less excessive shrinkage and lower dataset dependency. Although some additional simulation studies are required to evaluate performance, the *maxT* approach can be alternatively used. Finally, our permutation strategy requires an intensive computational burden to compute *p*-values. Thus, in future studies, we will derive a statistical distribution of the beta coefficient in *mimi-surv*, to avoid permutation procedures. Nonetheless, our *mimi-surv* remains promising for associating survival time with the expression of miRNAs and small non-coding RNAs whose misexpression is now widely accepted.

## Data Availability Statement

The data analyzed in this study is subject to the following licenses/restrictions: The datasets used in this study are provided upon the approval of individual data provider. Requests to access these datasets should be directed to J-YJ, jangjy4@gmail.com.

## Author Contributions

YK and TP: conceptualization and methodology. SuL: software. SeL, YK, and SuL: validation. SuL and YK: formal analysis and visualization. J-YJ: resources and data curation. YK: investigation and writing—original draft preparation. SuL and TP: writing—review and editing. TP: supervision, project administration, and funding acquisition. All authors contributed to the article and approved the submitted version.

## Conflict of Interest

The authors declare that the research was conducted in the absence of any commercial or financial relationships that could be construed as a potential conflict of interest.

## Footnotes

1 https://portal.gdc.cancer.gov/
