# Supplementary material for: Identifying miRNA-mRNA Integration Set Associated With Survival Time
Source: Front Genet. 2021 Jun 29;12:634922. doi: 10.3389/fgene.2021.634922 (PMC8276759; doi:10.3389/fgene.2021.634922)
Supplement: Supplementary file 1 [file Image_1.pdf]

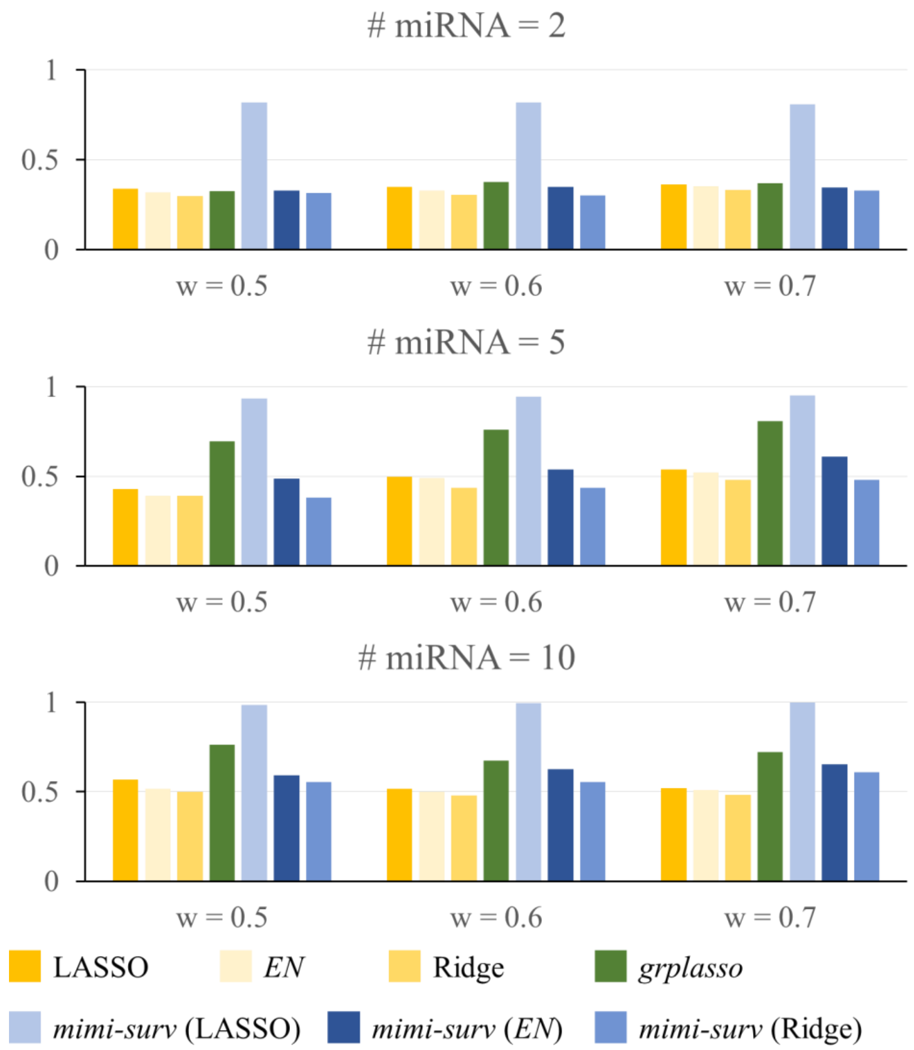

Supplementary Figure 1. Statistical powers of *mimi-surv* and the compared methods with different causal miRNA number and mRNA effect sizes (*w* = 0.2, 0.3 and 0.4). The phenotypes were generated from two, five and ten causal miRNA-mRNA integration set and censoring fraction of 0.3.
